# Novel haloarchaeon *Natrinema thermophila* having the highest growth temperature among haloarchaea with a large genome size

**DOI:** 10.1038/s41598-018-25887-7

**Published:** 2018-05-17

**Authors:** Yeon Bee Kim, Joon Yong Kim, Hye Seon Song, Changsu Lee, Seung Woo Ahn, Se Hee Lee, Min Young Jung, Jin-Kyu Rhee, Juseok Kim, Dong-Wook Hyun, Jin-Woo Bae, Seong Woon Roh

**Affiliations:** 1 Microbiology and Functionality Research Group, World Institute of Kimchi, Gwangju, 61755 Republic of Korea; 2 Department of Food Science and Engineering, Ewha Womans University, Seoul, 03760 Republic of Korea; 3 Department of Biology, Kyung Hee University, Seoul, 02447 Republic of Korea

## Abstract

Environmental temperature is one of the most important factors for the growth and survival of microorganisms. Here we describe a novel extremely halophilic archaeon (haloarchaea) designated as strain CBA1119^T^ isolated from solar salt. Strain CBA1119^T^ had the highest maximum and optimal growth temperatures (66 °C and 55 °C, respectively) and one of the largest genome sizes among haloarchaea (5.1 Mb). It also had the largest number of strain-specific pan-genome orthologous groups and unique pathways among members of the genus *Natrinema* in the class *Halobacteria*. A dendrogram based on the presence/absence of genes and a phylogenetic tree constructed based on OrthoANI values highlighted the particularities of strain CBA1119^T^ as compared to other *Natrinema* species and other haloarchaea members. The large genome of strain CBA1119^T^ may provide information on genes that confer tolerance to extreme environmental conditions, which may lead to the discovery of other thermophilic strains with potential applications in industrial biotechnology.

## Introduction

The growth of most microorganisms is influenced by physical factors such as temperature, water activity, pH, pressure, salinity, and oxygen concentration as well as chemical factors such as availability of nutrients (e.g., carbon and nitrogen)^1–4^. Microorganisms are usually classified based on optimal growth temperature-i.e., as psychrophiles, mesophiles, thermophiles, and hyperthermophiles, which grow best at temperatures of ≤15 °C, 15 °C–45 °C, >45 °C, and 80 °C, respectively^5^. These classes also differ in terms of the amino acid composition, structure, and thermostability of proteins^6^. Growth temperature seems to be related to genomic features; one study showed that the average length of proteins is shorter in thermophiles (growing best at temperatures of >45 °C) as compared to their homolog in mesophiles (15 °C–45 °C), whereas the proportion of purine bases in the coding strand is higher in the former than in the latter^7^. Other environmental factors besides temperature affect genome size: for example, the small genomes of prokaryotes are thought to reflect adaptation to strong selective pressures in large microbial populations, while the genome size in geophytes was found to be positively correlated with early flowering and growth tendency under humid conditions^8,9^.

Extremely halophilic archaea (haloarchaea) belonging to the domain Archaea are usually found in hypersaline environments such as salt lakes and crystallizer ponds from artificial marine solar salterns and in salty fermented foods and salted hides^10,11^, as well as in avian feather^12^. The growth temperature of haloarchaeal type strains ranges from −1 °C to 62 °C, with few growing at temperatures >60 °C (see Supplementary information). Genus *Natrinema* in the family Natrialbaceae includes eight known species of haloarchaea: *Natrinema altunense*, *Nnm. ejinorense*, *Nnm. gari*, *Nnm. pallidum*, *Nnm. pellirubrum*, *Nnm. salaciae*, *Nnm. soli*, and *Nnm. versiforme*^13–19^. In this study we describe strain CBA1119^T^ isolated from solar salt, which has the highest growth temperature and one of the largest genome sizes among all of the haloarchaeal members. We identified and characterized thermophilic strain CBA1119^T^ and investigated the relationship between two strain-specific features, namely growth temperature and genome size.

## Results and Discussion

**Figure 1:**
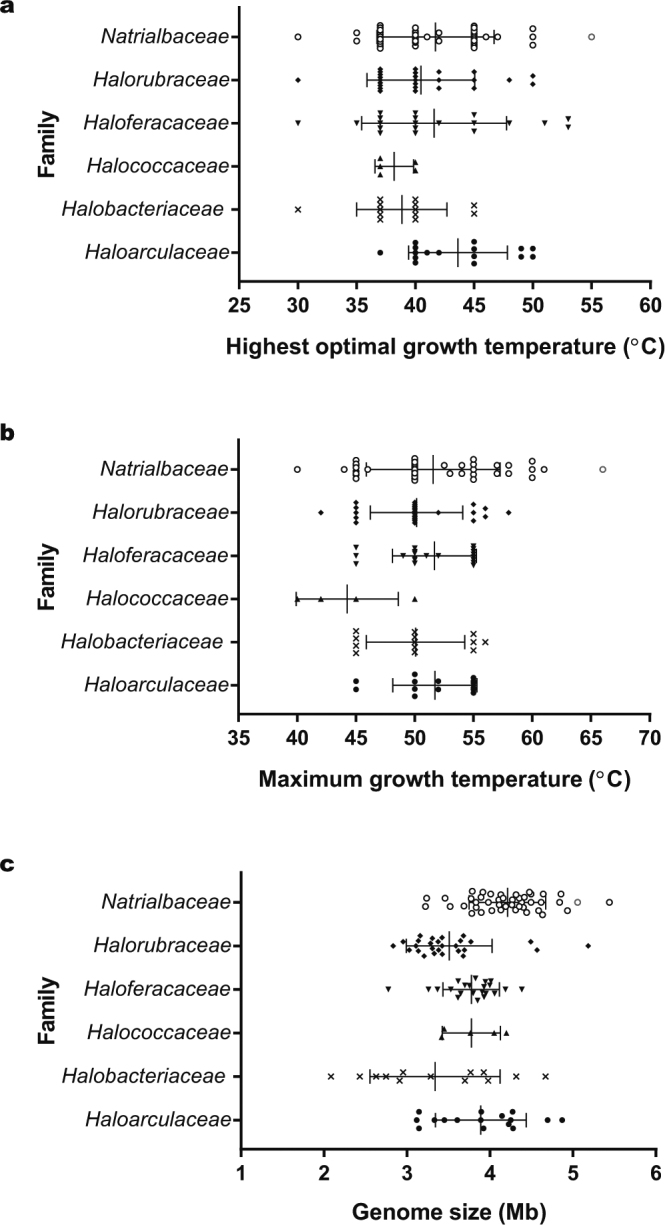
Comparison of the highest optimal (**a**) and maximum growth temperatures (**b**), and genome sizes (**c**) among haloarchaeal species. Strain CBA1119^T^ has the highest optimal and maximum growth temperature, and the third largest genome size among type strains belonging to haloarchaea. Red circles indicate strain CBA1119^T^.

Polyphasic taxonomic analysis (see Supplementary Information) revealed that strain CBA1119^T^ belonged to the genus *Natrinema* and was a novel member of the genus *Natrinema*. Interestingly, strain CBA1119^T^ grew at a temperature of 20 °C–66 °C; optimal growth was observed at 50 °C–55 °C. Of the four strains with an optimal growth temperature >50 °C; three belonged to the family Haloferacaceae and one was strain CBA1119^T^, which belongs to the family Natrialbaceae (Fig. 1a). The maximum growth temperatures of haloarchaea varied within each family (Fig. 1b). It is worth noting that there were no strains belonging to the family Halococcaceae that grew at temperatures >50 °C, and only those belonging to the family Natrialbaceae had a maximum growth temperature >60 °C, including strain CBA1119^T^. The maximum and optimal growth temperatures of strain CBA1119^T^ were the highest recorded to date among haloarchaea. Environmental temperature underlies the evolution of various biological phenomena such as the density of hydrogen bonds in nucleic acid^20^.

**Figure 2:**
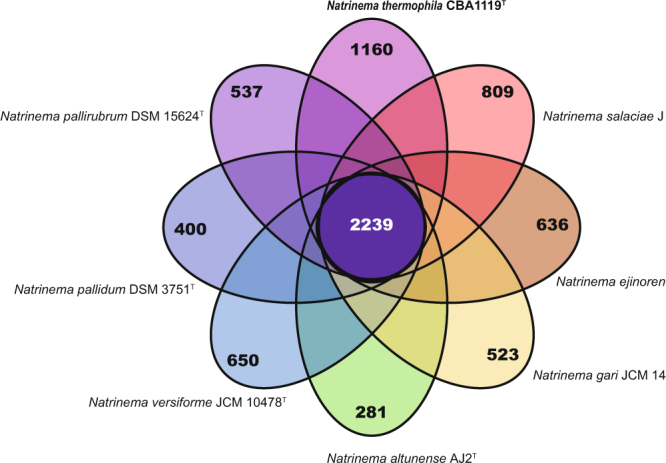
Flower plot showing strain-specific and core POGs of eight *Natrinema* species.

**Figure 3:**
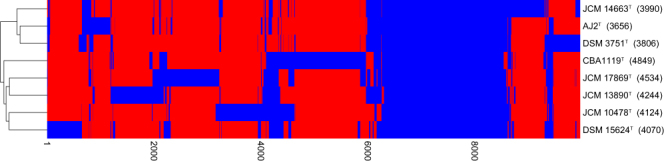
Heatmap based on gene content. A dendrogram was generated using Jaccard coefficients and unweighted pair-group method with arithmetic mean clustering. Blue and red indicate present and absent genes, respectively. Values in the brackets indicate number of POGs of each strain.

General genomic features of strain CBA1119^T^ were described in Supplementary Information (Supplementary Tables S2 and S3; Supplementary Fig. S2b). The ways in which the microbial genome is affected by environmental factors can be understood by pan-genome comparisons^21^. The number of pan-genome orthologous groups (POGs) and strain-specific POGs (singletons) were compared among strain CBA1119^T^ and seven species of the genus *Natrinema* (Fig. 2). The flower plot showed that strain CBA1119^T^ had the largest number of singletons among *Natrinema* species. The number of singletons in strain CBA1119^T^ was 1.4 times that in *Nnm. salaciae* JCM 17869^T^ (which had the second largest number) and four times that in *Nnm. altunense* AJ2^T^ (which had the smallest number). The heat map based on gene content also showed that strain CBA1119^T^ had more exclusive POGs than other related species (Fig. 3). Additionally, each genome within the genus *Natrinema* had distinct KEGG pathway profiles based on POGs (Table 1). Strain CBA1119^T^ had specific enzymes listed on the KEGG pathway named propanoate metabolism, geraniol degradation, fatty acid biosynthesis, metabolism and degradation, and valine, leucine and isoleucine degradation, with P values of zero.

**Table 1:** Strain-specific POGs listed on the KEGG pathway (P < 0.05).

Strain	KEGG pathway ID	Pathway name	Differentially present POGs	P value
CBA1119^T*^	MAP00281	Geraniol degradation	13	0.0000
	MAP00640	Propanoate metabolism	31	0.0000
	MAP00061	Fatty acid biosynthesis	16	0.0000
	MAP01212	Fatty acid metabolism	36	0.0000
	MAP00071	Fatty acid degradation	26	0.0000
	MAP00280	Valine, leucine, and isoleucine degradation	28	0.0000
	MAP00780	Biotin metabolism	12	0.0001
	MAP00072	Synthesis and degradation of ketone bodies	8	0.0001
	MAP00650	Butanoate metabolism	24	0.0002
	MAP01040	Biosynthesis of unsaturated fatty acids	12	0.0011
	MAP00380	Tryptophan metabolism	18	0.0026
	MAP00410	beta-Alanine metabolism	13	0.0032
	MAP00930	Caprolactam degradation	7	0.0064
	MAP00903	Linonene and pinene degradation	7	0.0168
	MAP00310	Lysine degradation	16	0.0173
	MAP00620	Pyruvate metabolism	18	0.0295
	MAP03022	Basal transcription factors	9	0.0408
	MAP00720	Carbon fixation pathways in prokaryotes	21	0.0414
JCM 17869^T^	MAP00072	Synthesis and degradation of ketone bodies	4	0.0101
JCM 13890^T^	MAP00780	Biotin metabolism	5	0.0180
	MAP00625	Chloroalkane and chloroalkene degradation	6	0.0298
	MAP00633	Nitrotoluene degradation	4	0.0394
JCM 14663^T^	MAP00072	Synthesis and degradation of ketone bodies	4	0.0033
JCM 10478^T^	MAP00983	Drug metabolism – other enzymes	5	0.0304
DSM 3751^T^	MAP00250	Alanine, aspartate and glutamate metabolism	7	0.0356
	MAP00650	Butanoate metabolism	8	0.0363
DSM 15624^T^	MAP03022	Basal transcription factors	7	0.0067

^*^Strain CBA1119^T^ is estimated to contain the largest number of singletons.

For genome size and growth temperature comparisons among haloarchaeal type strains, information on the strains was obtained from the GenBank database and previous studies, and is shown in Supplementary Table S4. Genome size comparison at the class level revealed that most haloarchaea (104/128 species) had a genome ranging between 3.0 and 4.5 Mb in size, with the class *Natrialbaceae* having the largest average genome size (Fig. 1c). Interestingly, only three strains had a genome size >5 Mb, including strain CBA1119^T^. Besides a high growth temperature, strain CBA1119^T^ had an unusually large genome size. Haloarchaea species with a genome >5 Mb are uncommon; only two such type strains (and three in total) are found in the GenBank database. Strain CBA1119^T^ had the third largest genome among haloarchaeal type strains (and the fourth among total haloarchaeal strains). Genome size was shown to be related to COG categories and pathways in bacteria; COG categories related to secondary metabolism and energy conversion were more highly represented in larger genomes, as were KEGG categories related to various cellular processes and metabolism with the exception of nucleotide metabolism^22^. Free-living bacteria with a genome size >6 Mb such as *Bacteroides thetaiotaomicron* and *Streptomyces avermitili* can grow in various environments and use a wide range of substrates for energy production. Thus, strain CBA1119^T^ with its large genome size may be capable of growing under different conditions, and can potentially utilize different substrates to produce energy. Genome size increases with the level of environmental instability; that is, large genomes are also more resistant to environmental perturbations than smaller ones^23^. It remains to be determined whether this applies to strain CBA1119^T^. Clarifying the genomic and environmental factors that affect growth temperature and genome size can provide insight into environment-microbe interactions and evolutionary adaptations of various microorganisms, while additional studies on the enzymes of strain CBA1119^T^ can reveal new tools for industrial biotechnology applications.

## Materials and Methods

### Isolation of archaeal strain

Strain CBA1119^T^ was isolated from unrefined solar salt obtained from a salt field (34.587738 N, 126.105372 E) in the Republic of Korea and aerobically cultured in DBCM2 medium (JCM medium no. 574; 833 ml MDS salt water [240 g NaCl, 30 g MgCl_2_∙6H_2_O, 35 g MgSO_4_∙7H_2_O, 7 g KCl, 5 ml 1 M CaCl_2_ solution per liter], 1 ml FeCl_2_ solution [10 ml 25% HCl, 1.5 g FeCl_2_∙4H_2_O per liter], 1 ml trace element solution [70 mg ZnCl_2_, 100 mg MnCl_2_∙4H_2_O, 6 mg H_3_BO_3_, 190 mg CoCl_2_∙6H_2_O, 2 mg CuCl_2_∙2H_2_O, 24 mg NiCl_2_∙6 H_2_O, 36 mg Na_2_MoO_4_∙2H_2_O per liter], 0.25 g peptone [Oxoid, Chesire, UK], 0.05 g yeast extract [BD Biosciences, Franklin Lakes, NJ, USA], 5 ml 1 M NH_4_Cl, 3 ml vitamin solution [3 mg biotin, 4 mg folic acid, 50 mg pyridoxine·HCl, 33 mg thiamine·HCl, 10 mg riboflavin, 33 mg nicotinic acid, 17 mg DL-calcium pantothenate, 17 mg vitamin B12, 13 mg para-aminobenzoic acid, 10 mg lipoic acid per liter], 10 ml of 1 M sodium pyruvate solution, 2 ml potassium phosphate buffer [417 ml 1 M K_2_HPO_4_ and 83 ml 1 M KH_2_PO_4_ per liter], and 50 ml 1 M Tris-HCl, pH 7.5 per liter) at 37 °C for 4 weeks. To obtain pure culture, a single colony was transferred repeatedly to the agar medium.

### Phenotypic, chemotaxonomic, and phylogenetic analyses

Phenotypic tests were performed according to the minimal standards for description of new taxa in the order *Halobacteriales*^24^. Cell morphology and size were examined by field emission transmission electron microscopy (Chuncheon Center, Korea Basic Science Institute, Korea). Gram staining was performed as previously described^25^. For comparative phenotypic analyses, reference strains were selected based on the relatedness of 16S rRNA gene sequences (>97%). For this purpose, *Nnm. soli* LMG 29247^T18^, *Nnm. salaciae* JCM 17869^T17^, and *Nnm. ejinorense* JCM 13890^T14^ obtained from the Japan Collection of Microorganisms (JCM) or Belgian Coordinated Collections of Microorganisms (BCCM) were cultured at 37 °C in DBCM2 medium. Growth at different temperatures (4 °C, 15 °C–60 °C at intervals of 5 °C, and 61 °C–70 °C at intervals of 1 °C), NaCl concentrations (0–30% [w/v] at intervals of 5%), pHs (5.0–11.0 at intervals of 1.0), and Mg^2+^ concentrations (0, 5, 10, 20, 50, 100, 200, and 500 mM) were tested using DBCM2 medium as the basal medium for 4 weeks. pH was adjusted by adding the following buffers: 10 mM 2-(N-morpholino)-ethanesulfonic acid (MES) (pH 5–6), 1,3-bis[tris(hydroxymethyl)methylamino]propane (Bis-TRIS propane) (pH 7–9), or N-cyclohexyl-3-aminopropanesulfonic acid (CAPS) (pH 10–11). Anaerobic growth in the presence of 0.5% l-arginine, trimethylamine-N-oxide (TMAO), dimethyl sulfoxide (DMSO), or 30 mM nitrate was evaluated on DBCM2 medium at 37 °C in an anaerobic chamber (Coy Laboratory Products, Grass Lake, MI, USA) with an N_2_·CO_2_·H_2_ (90:5:5, v-v:v) atmosphere. Catalase and oxidase activities^26^ as well as the hydrolysis of starch and casein^27^ and of Tween 40 and Tween 80^28^ were evaluated according to established protocols. Antibiotic susceptibility was tested on DBCM2 medium using antibiotic discs with ampicillin (10 μg per disc), erythromycin (15 μg), gentamicin (10 μg), kanamycin (30 μg), nalidixic acid (30 μg), rifampicin (10 μg), and streptomycin (10 μg). The effectiveness of various substrates as a sole carbon and energy source and acid production were determined in HMD medium^29^. A total of 20 carbon sources were tested: D-fructose, D-galactose, D-mannitol, D-mannose, D-sorbitol, D-xylose, fumarate, glycerol, maltose, pyruvate, starch, succinate, sucrose, L-alanine, L-arginine, L-aspartate, L-glutamate, L-lysine, L-malate, and L-sorbose. Polar lipids from strain CBA1119^T^ were extracted, analyzed, and compared with those of the three reference strains as previously described^30^. The DNA-DNA hybridization (DDH)^31^ was performed to determine the genetic relationship between strain CBA1119^T^ and the three reference strains. To determine the taxonomic identity based on 16S rRNA gene sequence, chromosomal DNA was extracted using a commercial DNA extraction kit (iNtRON Biotechnology, Sungnam, Korea) and the 16S rRNA gene was amplified using PCR PreMix (iNtRON Biotechnology) with universal primers 0018 F and 1518R^32^. Amplified 16S rRNA PCR products were sequenced and assembled as previously described^33^ and 16S rRNA sequences were compared using EzTaxon-e^34^ or NCBI BLAST^35^. Phylogenetic trees were constructed based on the three 16S rRNA gene sequences of strain CBA1119^T^ obtained from the genome sequencing data (see below) and other related species using MEGA6 software^36^. Phylogenetic trees were generated with neighbor-joining (NJ)^37^, maximum likelihood (ML)^38^, and maximum parsimony (MP)^39^ methods with 1 000 bootstrap replications based on the NJ tree.

### Library preparation, sequencing, genome assembly, and annotation

To clarify the relationship between physiological characteristics (especially capacity for growth at high temperatures) and genomic features, we performed genome sequencing of strain CBA1119^T^ and *Nnm. ejinorense* JCM 13890^T^ as previously described^40^. In brief summary, the genomic DNA shearing and SMRTbell library preparation were carried out according to the standard PacBio 20-kb Template Preparation Using BluePippin Size-Selection System protocol by P6-C4 chemistry (Pacific Biosciences, Menlo Park, CA, USA), respectively. The strain CBA1119^T^ genome and *Nnm. ejinorense* JCM 13890^T^ genome sequences were determined using the PacBio RS II system (Pacific Biosciences). *De novo* genome assembly of each genome was performed using Hierarchical Genome Assembly Process v.2 software with default parameters supported by PacBio SMRT Analysis v.2.3.0^41^. rRNA and tRNA prediction was carried out using RNAmmer v.1.2^42^ and tRNAscan-SE v.1.21^43^, respectively. Genes were predicted using Glimmer3 in Rapid Annotation using Subsystem Technology server (http://rast.nmpdr.org), and functional gene annotations were performed based on the SEED, COG (http://www.ncbi.nlm.nih.gov/COG), and KEGG (http://www.genome.jp/kegg/) databases. The GenBank/EMBL/DDBJ accession numbers for the *Natrinema thermophila* CBA1119^T^ and *Natrinema ejinorense* JCM 13890^T^ are PDBS00000000 and NXNI00000000, respectively.

### Comparative genomic analysis

For genomic comparisons, *Natrinema* species genomes were obtained from the NCBI genome database, except those of strains CBA1119^T^ and JCM 13890^T^, which were sequenced as described above. The OrthoANI algorithm was used to analyze the genomic relatedness between strain CBA1119^T^ and other species. OrthoANI percentages were calculated and a phylogenetic tree was constructed^44^. Orthologs in strain CBA1119^T^ and the reference strains were predicted and mapped using the reciprocal best hit method in UBLAST^45^. Pan-genome orthologous groups (POGs) were estimated using the EzBioCloud Comparative Genomics Database (http://cg.ezbiocloud.net/)^46^, and their presence was calculated using the Jaccard coefficient. The unweighted pair-group method with arithmetic mean (UPGMA) clustering was then used to assess clustering between strain CBA1119^T^ and the reference strains from a dendrogram constructed based on the presence or absence of gene content. Haloarchaea genomes for comparisons were obtained from the NCBI genome database according to the following criteria: genomes with optimal or maximum growth temperature information were selected for comparisons of optimal and maximum growth temperature, respectively; genomes of unclassified strains^47^ were excluded; and genomes with fewer contigs that are less incomplete were selected, when multiple genomes were available for a single strain.

## Electronic supplementary material

Supplementary Information

## References

[1] Baez A, Shiloach J (2014). Effect of elevated oxygen concentration on bacteria, yeasts, and cells propagated for production of biological compounds. Microb Cell Fact.

[2] Engelkirk, P. G., Duben-Engelkirk, J. L. & Burton, G. R. W. *Burton’s microbiology for the health sciences*. Lippincott Williams & Wilkins (2011).

[3] Forbort, J. Treatment of Organic Contaminants: Biological Treatment. In: *Groundwater Treatment Technology*. John Wiley & Sons, Inc. (2009).

[4] Mota MJ, Lopes RP, Delgadillo I, Saraiva JA (2013). Microorganisms under high pressure — Adaptation, growth and biotechnological potential. Biotechnol Adv.

[5] Madigan, M. T., Martinko, J. M., Bender, K. S., Buckley, D. H. & Stahl, D. A. *Brock Biology of Microorganisms*, 14th edn. Pearson Education Limited (2015).

[6] Dworkin, M., Falkow, S., Rosenberg, E., Schleifer, K. H. & Stackebrandt, E. *The Prokaryotes: Vol. 2: Ecophysiology and Biochemistr*y. Springer New York (2006).

[7] Hickey DA, Singer GA (2004). Genomic and proteomic adaptations to growth at high temperature. Genome Biol.

[8] Sela I, Wolf YI, Koonin EV (2016). Theory of prokaryotic genome evolution. Proc Natl Acad Sci USA.

[9] Vesely P, Bures P, Smarda P, Pavlicek T (2012). Genome size and DNA base composition of geophytes: the mirror of phenology and ecology?. Ann Bot.

[10] Oren, A. The Order *Halobacteriales*. In: *The Prokaryotes: Volume 3: Archaea. Bacteria: Firmicutes, Actinomycetes*. Springer New York (2006).

[11] Roh SW (2009). Investigation of archaeal and bacterial diversity in fermented seafood using barcoded pyrosequencing. ISME J.

[12] Yim KJ (2015). Occurrence of viable, red-pigmented haloarchaea in the plumage of captive flamingoes. Sci Rep.

[13] Xu X-W, Ren P-G, Liu S-J, Wu M, Zhou P-J (2005). *Natrinema altunense* sp. nov., an extremely halophilic archaeon isolated from a salt lake in Altun Mountain in Xinjiang, China. Int J Syst Evol Microbiol.

[14] Castillo AM (2006). *Natrinema ejinorense* sp. nov., isolated from a saline lake in Inner Mongolia, China. Int J Syst Evol Microbiol.

[15] Tapingkae W (2008). *Natrinema gari* sp. nov., a halophilic archaeon isolated from fish sauce in Thailand. Int J Syst Evol Microbiol.

[16] McGenity TJ, Gemmell RT, Grant WD (1998). Proposal of a new halobacterial genus *Natrinema* gen. nov., with two species *Natrinema pellirubrum* nom. nov. and *Natrinema pallidum* nom. nov. Int J Syst Bacteriol.

[17] Albuquerque L, Taborda M, La Cono V, Yakimov M, da Costa MS (2012). *Natrinema salaciae* sp. nov., a halophilic archaeon isolated from the deep, hypersaline anoxic Lake Medee in the Eastern Mediterranean Sea. Syst Appl Microbiol.

[18] Rasooli M (2017). *Natrinema soli* sp. nov., a novel halophilic archaeon isolated from a hypersaline wetland. Int J Syst Evol Microbiol.

[19] Xin H (2000). *Natrinema versiforme* sp. nov., an extremely halophilic archaeon from Aibi salt lake, Xinjiang, China. Int J Syst Evol Microbiol.

[20] Jeffrey, G. A. & Saenger, W. *Hydrogen Bonding in Biological Structures*. Springer Berlin Heidelberg (2012).

[21] Tettelin H, Riley D, Cattuto C, Medini D (2008). Comparative genomics: the bacterial pan-genome. Curr Opin Microbiol.

[22] Konstantinidis KT, Tiedje JM (2004). Trends between gene content and genome size in prokaryotic species with larger genomes. Proc Natl Acad Sci USA.

[23] Bentkowski P, Van Oosterhout C, Mock T (2015). A Model of Genome Size Evolution for Prokaryotes in Stable and Fluctuating Environments. Genome Biol Evol.

[24] Oren A, Ventosa A, Grant WD (1997). Proposed Minimal Standards for Description of New Taxa in the Order *Halobacteriales*. Int J Syst Bacteriol.

[25] Dussault HP (1955). An improved technique for staining red halophilic bacteria. J Bacteriol.

[26] Benson, H. J. Microbiological applications: a laboratory manual in general microbiology. McGraw-Hill Higher Education Boston (2002).

[27] Smibert, R. M. & Kreg, N. R. Phenotypic characterization. In: Methods for General and Molecular Bacteriology. Gerhardt, P., Murray, R. G. E., Wood, W. A. & Krieg, N. R. Eds *American Society for Microbiology, Washington, DC*, 607–654 (1994).

[28] Gonzalez C, Gutierrez C, Ramirez C (1978). *Halobacterium vallismortis* sp. nov. An amylolytic and carbohydrate-metabolizing, extremely halophilic bacterium. Can J Microbiol.

[29] Savage KN, Krumholz LR, Oren A, Elshahed MS (2007). *Haladaptatus paucihalophilus* gen. nov., sp. nov., a halophilic archaeon isolated from a low-salt, sulfide-rich spring. Int J Syst Evol Microbiol.

[30] Minnikin, D. E. *et al*. An integrated procedure for the extraction of bacterial isoprenoid quinones and polar lipids. *J Microbiol Methods*, 233–241 (1984).

[31] Ezaki T, Hashimoto H, Yabuuchi E (1989). Fluorometric deoxyribonucleic acid-deoxyribonucleic acid hybridization in microdilution wells as an alternative to membrane filter hybridization in which radioisotopes are used to determine genetic relatedness among bacterial strains. Int J Syst Bacteriol.

[32] Cui HL, Zhou PJ, Oren A, Liu SJ (2009). Intraspecific polymorphism of 16S rRNA genes in two halophilic archaeal genera, *Haloarcula* and *Halomicrobium*. Extremophiles.

[33] Roh SW (2008). *Arthrobacter soli* sp. nov., a novel bacterium isolated from wastewater reservoir sediment. J Microbiol.

[34] Kim OS (2012). Introducing EzTaxon-e: a prokaryotic 16S rRNA gene sequence database with phylotypes that represent uncultured species. Int J Syst Evol Microbiol.

[35] Altschul SF, Gish W, Miller W, Myers EW, Lipman DJ (1990). Basic local alignment search tool. J Mol Biol.

[36] Tamura K, Stecher G, Peterson D, Filipski A, Kumar S (2013). MEGA6: Molecular Evolutionary Genetics Analysis version 6.0. Mol Biol Evol.

[37] Saitou N, Nei M (1987). The neighbor-joining method: a new method for reconstructing phylogenetic trees. Mol Biol Evol.

[38] Felsenstein J (1981). Evolutionary trees from DNA sequences: a maximum likelihood approach. J. Mol. Evol.

[39] Fitch WM (1971). Toward defining the course of evolution: minimum change for a specific tree topology. Syst Zool.

[40] Kim JY (2016). Genome sequence of a commensal bacterium, *Enterococcus faecalis* CBA7120, isolated from a Korean fecal sample. Gut Pathog.

[41] Chin CS (2013). Nonhybrid, finished microbial genome assemblies from long-read SMRT sequencing data. Nat Methods.

[42] Lagesen K (2007). RNAmmer: consistent and rapid annotation of ribosomal RNA genes. Nucleic Acids Res.

[43] Lowe TM, Eddy SR (1997). tRNAscan-SE: a program for improved detection of transfer RNA genes in genomic sequence. Nucleic Acids Res.

[44] Lee I, Ouk Kim Y, Park SC, Chun J (2016). OrthoANI: An improved algorithm and software for calculating average nucleotide identity. Int J Syst Evol Microbiol.

[45] Ward N, Moreno-Hagelsieb G (2014). Quickly finding orthologs as reciprocal best hits with BLAT, LAST, and UBLAST: how much do we miss?. PLoS One.

[46] Yoon SH (2017). Introducing EzBioCloud: a taxonomically united database of 16S rRNA gene sequences and whole-genome assemblies. Int J Syst Evol Microbiol.

[47] Gupta RS, Naushad S, Fabros R, Adeolu M (2016). A phylogenomic reappraisal of family-level divisions within the class *Halobacteria*: proposal to divide the order *Halobacteriales* into the families *Halobacteriaceae*, *Haloarculaceae* fam. nov., and *Halococcaceae* fam. nov., and the order *Haloferacales* into the families, *Haloferacaceae* and *Halorubraceae* fam nov. Antonie Van Leeuwenhoek.

