# Supplementary material for: Novel haloarchaeon Natrinema thermophila having the highest growth temperature among haloarchaea with a large genome size
Source: Sci Rep. 2018 May 17;8:7777. doi: 10.1038/s41598-018-25887-7 (PMC5958107; doi:10.1038/s41598-018-25887-7)
Supplement: Supplementary file 1 — Supplementary Information [file 41598_2018_25887_MOESM1_ESM.pdf]

Supplementary Information

Novel haloarchaeon *Natrinema thermophila* having the highest growth temperature among haloarchaea with a large genome size

Yeon Bee Kim, Joon Yong Kim, Hye Seon Song, Changsu Lee, Seung Woo Ahn, Se Hee Lee, Min Young Jung, Jin-Kyu Rhee, Juseok Kim, Dong-Wook Hyun, Jin-Woo Bae and Seong Woon Roh

Polyphasic taxonomic analysis

Strain CBA1119^{T} was coccoid and 0.9–1.0 µm in size, and grew at an NaCl concentration of 10%–30% (w/v) and at pH 7–9 and a temperature of 20 °C–66 °C. Optimum growth was observed at 20%–25% (w/v) of NaCl, pH 7–8, and 50 °C–55 °C. Mg^{2+} was not required for growth. Under anaerobic conditions, no growth occurred in the presence of L-arginine, trimethylamine-N-oxide (TMAO), dimethylsulfoxide (DMSO), or nitrate. Strain CBA1119^{T} was positive for catalase and oxidase activities and for hydrolysis of starch, Tween 40, and Tween 80, but was negative for the hydrolysis of casein. Strain CBA1119^{T} was sensitive to rifampicin but not to cycloheximide, erythromycin, kanamycin, nalidixic acid, streptomycin, or vancomycin. Detailed characteristics are summarized in Supplementary Table S1 and the species description below. The major polar lipids of strain CBA1119^{T} were phosphatidylglycerol (PG), phosphatidylglycerol phosphate methyl ester (PGP-Me), phosphatidylglycerol sulfate (PGS), unidentified phospholipid (PL), unidentified glycolipids (GL1 and GL2) and unidentified lipid (L1) (Supplementary Figure S1). Strain CBA1119^{T} had 98.8% of 16S rRNA gene sequence similarities with *Natrinema soli* 5-3^{T}, 97.1–97.2% with

Nnm. salaciae MDB25^{T}, and 97.0% with *Nnm. ejinorense* EJ-57^{T}, followed by < 97% with other haloarchaeal members. DNA-DNA hybridization (DDH) values between strain CBA1119^{T} and the reference strains: *Natrinema soli* LMG 29247^{T}, *Nnm. salaciae* JCM 17869^{T} and *Nnm. ejinorense* JCM 13890^{T}, were 37.0%, 30.6% and 23.4%, respectively. The low DDH value (< 70%)[1] indicated that strain CBA1119^{T} is a distinct species within the genus *Natrinema*. It is known that the genera *Natrinema* and *Haloterrigena* show a very close relationship as not forming each monophyletic group at the genus level in phylogenetic trees[2-5]. The phylogenetic tree based on 16S rRNA gene similarities showed that strain CBA1119^{T} clustered tightly with the type strain of *Natrinema soli*, implying that strain CBA1119^{T} is more closely related to the member in the genus *Natrinema* than other genus members (Supplementary Figure S2a). The polyphasic taxonomic data indicate that strain CBA1119^{T} represents a novel species of the genus *Natrinema*, for which the name *Natrinema thermophila* is proposed.

Description of Natrinema thermophila sp. nov.

Natrinema thermophila (ther.mo'phi.la. Gr. n. *thermê* heat; N.L. fem. adj. *phila* (from Gr. fem. adj. *philê*) loving; N.L. fem. adj. *thermophila* heat-loving organism).

The cells were coccoid in shape and Gram-negative. Colonies were orange when grown in DBCM2 medium. Growth occurred at 20 °C–66 °C (optimum, 50 °C–55 °C), with 10%–30% (w/v) NaCl (optimum, 20%–25%), and at pH 7–9 (optimum, pH 7–8). Mg^{2+} was not required for growth. Strain CBA1119^{T} did not grow anaerobically in the presence of nitrate, L-arginine, DMSO, or TMAO, and was catalase- and oxidase-positive. The strain was positive for hydrolysis of starch, Tween 40, and Tween 80 and negative for hydrolysis of casein. D-Sorbitol, fumarate, glycerol, L-glutamate, L-malate, pyruvate, starch, succinate, and sucrose were utilized as sole sources of carbon and energy, while D-fructose, D-galactose, D-mannitol, D-mannose, D-xylose, D-maltose, L-alanine, L-arginine, L-aspartate, L-lysine, and L-sorbose were

not. The major polar lipids were phosphatidylglycerol phosphate methyl ester and phosphatidylglycerol sulfate, and the G+C content was 62.27 mol%. The type strain was CBA1119ᵀ (= KCCM 43222ᵀ = JCM 30478ᵀ), which was isolated from solar salt from a salt field in the Republic of Korea.

General genomic features of strain CBA1119ᵀ

The genome of strain CBA1119ᵀ comprised nine contigs that were 5 058 058 bp long with a G+C content of 62.27 mol%; one of the contigs was a chromosome that was 4 087 412 bp long and had a G+C content of 63.4 mol%. The genome had 5 122 open reading frames, 10 rRNA genes (three 16S, three 23S, and four 5S rRNA), and 49 tRNA genes. A total of 4 482 genes were annotated to Clusters of Orthologous Groups (COG); among them, 2 508 were matched to these 16 functional categories as shown in Supplementary Table S2. Additionally, 1 974 genes were matched to the S (function unknown) category. The range of OrthoANI values between strain CBA1119ᵀ and other type strains of the genus *Natrinema* was 81.93%–82.94% (Supplementary Table S3). The phylogenetic tree constructed from these values indicated that strain CBA1119ᵀ was located as an outgroup (Supplementary Figure S2b). Members of the genus *Natrinema* were identified as non-human pathogens using PathogenFinder v.1.1 and had no antimicrobial resistance genes based on ResFinder v.2.1.

Supplementary Table S1. Differences in characteristics of strain CBA1119^{T} and related species[†].

Characteristic	1	2	3	4
Cell morphology	Cocci	Oval	Pleomorphic	Pleomorphic
NaCl optimum (% [w/v])	20.0−25.0	23.4	15.2−19.9	19.9
$MgCl_2$ requirement	−	−	−	−
Temperature for growth				
Range (°C)	20–66	25–45	30–52.5	25–50
Optimum (°C)	50–55	40	45	37
pH for growth				
Range	7.0–9.0	6.0–8.0	6.5–9.0	6.0–8.5
Optimum	7.0–8.0	7.0	7.0–8.0	7.0
Anaerobic growth of nitrate	−	−	+	−
Hydrolysis				
Starch	+	−	+	+
Casein	−	−	+	+
Tween 80	+	+	−	−
Assimilation				
D-Sorbitol	+	+	−	−
D-Maltose	−	+	+	+
Glycerol	+	+	−	−[*]
L-Alanine	−	+[*]	+	+
L-Arginine	−	+[*]	+	+

L-Aspartate	−	+	+	+
L-Lysine	−	+*	+	+
Acid production from				
Starch	−	−	+	+
Sucrose	−	+*	+	−
DNA G+C content (mol%)	62.27	61.5	64.6	64.7

Taxa: 1, *Natrinema thermophila* CBA1119^{T} sp. nov.; 2, *Nnm. soli* LMG 29247^{T4}; 3, *Nnm. salaciae* JCM 17869^{T2}; 4, *Nnm. ejinorense* JCM 13890^{T6}.

†Based on the data from this study, strain CBA1119^{T}, *Nnm. soli* LMG 29247^{T}, *Nnm. salaciae* JCM 17869^{T}, and *Nnm. ejinorense* JCM 13890^{T} all utilize fumarate, pyruvate, starch, succinate, sucrose, L-glutamate, and L-malate and could not utilize D-fructose, D-galactose, D-mannitol, D-mannose, and D-xylose. None of the strains produced acid from fumarate, succinate, L-glutamate, or L-malate. +, Positive; −, negative. Hydrolysis test, single carbon source assimilation, and acid production data are from this study.

*Opposite results were reported by original studies.

Supplementary Table S2. Genes associated with general COG functional categories in the genome of *Natrinema thermophila* CBA1119^T.

COG	Description of function	Number of genes	Ratio
J	Translation, ribosomal structure and biogenesis	161	3.59%
K	Transcription	237	5.29%
L	Replication, recombination and repair	318	7.10%
D	Cell cycle control, cell division, chromosome partitioning	21	0.47%
O	Posttranslational modification, protein turnover, chaperones	132	2.95%
M	Cell wall/membrane/envelope biogenesis	114	2.54%
N	Cell motility	17	0.38%
P	Inorganic ion transport and metabolism	229	5.11%
T	Signal transduction mechanisms	151	3.37%
C	Energy production and conversion	273	6.09%
G	Carbohydrate transport and metabolism	118	2.63%
E	Amino acid transport and metabolism	282	6.29%
F	Nucleotide transport and metabolism	77	1.72%
H	Coenzyme transport and metabolism	126	2.81%
I	Lipid transport and metabolism	179	3.99%
Q	Secondary metabolites biosynthesis, transport and catabolism	73	1.63%
R	General function prediction only	0	0.00%
S	Function unknown	1 974	44.04%
Total		4 482	100%

Supplementary Table S3. OrthoANI values between type strains in the genus *Natrinema*[†].

Strains	1	2	3	4	5	6	7	8
**1**	100.00	82.93	82.47	82.09	82.80	82.94	82.68	81.93
**2**	82.93	100.00	83.82	82.98	83.61	83.47	83.39	82.31
**3**	82.47	83.82	100.00	83.61	84.22	83.20	83.60	82.22
**4**	82.09	82.98	83.61	100.00	92.79	83.15	91.79	83.01
**5**	82.80	83.61	84.22	92.79	100.00	83.84	93.01	83.19
**6**	82.94	83.47	83.20	83.15	83.84	100.00	83.28	82.94
**7**	82.68	83.39	83.60	91.79	93.01	83.28	100.00	83.52
**8**	81.93	82.31	82.22	83.01	83.19	82.94	83.52	100.00

Taxa: 1, *Natrinema thermophila* CBA1119^{T} (PDBS00000000); 2, *Nnm. salaciae* JCM 17869^{T} (FOFD00000000); 3, *Nnm. ejinorense* JCM 13890^{T} (NXNI00000000); 4, *Nnm. gari* JCM 14663^{T} (AOIJ00000000); 5, *Nnm. altunense* AJ2^{T} (JNCS00000000); 6, *Nnm. versiforme* JCM 10478^{T} (AOID00000000); 7, *Nnm. pallidum* DSM 3751^{T} (AOII00000000); 8, *Nnm. pellirubrum* DSM 15624^{T} (CP003372).

[†]Based on these results, OrthoANI values between strain CBA1119^{T} and other *Natrinema* type strains were in the range of 81.93%–82.94%.

Supplementary Table S4. Type strains used for genome size and growth temperature comparisons. Information on the strains was obtained from the NCBI database and previous studies.

Family	Species	Strain	Assembly accession number	Size (Mb)	Optimal temperature (℃)	Maximum temperature (℃)	Reference for growth temperature data
Haloarculaceae	*Halapricum salinum*	CBA1105	GCA_000755225.1	3.45149	37	45	[7]
Haloarculaceae	*Haloarcula amylolytica*	JCM 13557	GCA_000336615.1	4.22542	41	52	[8]
Haloarculaceae	*Haloarcula argentinensis*	DSM12282	GCA_000336895.1	4.14711	40	ND	[9]
Haloarculaceae	*Haloarcula hispanica*	CGMCC 1.2049	GCA_000223905.1	3.89000	40	50	[10]
Haloarculaceae	*Haloarcula japonica*	DSM 6131	GCA_000336635.1	4.28036	45	45	[11]
Haloarculaceae	*Haloarcula marismortui*	ATCC 43049	GCA_000011085.1	4.27464	50	ND	[12]
Haloarculaceae	*Haloarcula vallismortis*	DSM 3756	GCA_900106715.1	3.89664	49	55	[13]
Haloarculaceae	*Halomicrobium katesii*	DSM 19301	GCA_000379085.1	3.60777	40	50	[14]
Haloarculaceae	*Halomicrobium mukohataei*	DSM 12286	GCA_000023965.1	3.33235	45	52	[15]
Haloarculaceae	*Halomicrobium zhouii*	CGMCC 1.10457	GCA_900114435.1	4.25033	42	55	[16]
Haloarculaceae	*Halorhabdus tiamatea*	SARL4B	GCA_000470655.1	3.14616	45	55	[17]
Haloarculaceae	*Halorhabdus utahensis*	DSM 12940	GCA_000023945.1	3.11680	50	55	[18]
Haloarculaceae	*Halorientalis persicus*	IBRC-M 10043	GCA_900110215.1	4.86976	40	50	[19]
Haloarculaceae	*Halosimplex carlsbadense*	2-9-1	GCA_000337455.1	4.69489	40	50	[20]
Halobacteriaceae	*Haladaptatus cibarius*	D43	GCA_000710615.1	3.92672	37	50	[21]
Halobacteriaceae	*Haladaptatus litoreus*	CGMCC 1.7737	GCA_900156425.1	4.67171	40	55	[22]
Halobacteriaceae	*Haladaptatus paucihalophilus*	DX253	GCA_000376445.1	4.31754	30	45	[23]
Halobacteriaceae	*Halalkalicoccus jeotgali*	B3	GCA_000196895.1	3.69865	45	50	[24]
Halobacteriaceae	*Halalkalicoccus paucihalophilus*	DSM 24557	GCA_001593955.1	3.98041	37	50	[25]
Halobacteriaceae	*Halarchaeum acidiphilum*	JCM 16109	GCA_000474235.1	2.62897	37	45	[26]
Halobacteriaceae	*Haloarchaeobius iranensis*	KCTC 4048	GCA_900103505.1	3.76861	40	55	[27]

Halobacteriaceae	*Halobacterium jilantaiense*	CGMCC 1.5337	GCA_900110535.1	2.95279	40	55	[28]
Halobacteriaceae	*Halodesulfurarchaeum formicicum*	HSR6	GCA_001886955.1	2.08595	37	50	[29]
Halobacteriaceae	*Halovenus aranensis*	IBRC-M 10015	GCA_900100385.1	3.28712	40	50	[30]
Halobacteriaceae	*Natronomonas moolapensis*	8.8.11	GCA_000591055.1	2.91257	40	45	[31]
Halobacteriaceae	*Natronomonas pharaonis*	DSM 2160	GCA_000026045.1	2.74970	45	56	[13]
Halococcaceae	*Halococcus hamelinensis*	100A6	GCA_000336675.1	3.41907	37	ND	[32]
Halococcaceae	*Halococcus saccharolyticus*	DSM 5350	GCA_000336915.1	3.44970	37	42	[33]
Halococcaceae	*Halococcus salifodinae*	DSM 8989	GCA_000336935.1	4.19978	40	50	[34]
Halococcaceae	*Halococcus sediminicola*	CBA1101	GCA_000755245.1	3.76437	40	40	[35]
Halococcaceae	*Halococcus thailandensis*	JCM 13552	GCA_000336715.1	4.05243	37	45	[36]
Haloferacaceae	*Halobellus clavatus*	CGMCC 1.10118	GCA_900107195	3.75498	37	50	[37]
Haloferacaceae	*Halobellus rufus*	CBA1103	GCA_000739575.1	3.85222	37	55	[38]
Haloferacaceae	*Haloferax alexandrinus*	JCM 10717	GCA_000336735.1	3.64926	ND	55	[39]
Haloferacaceae	*Haloferax denitrificans*	ATCC 35960	GCA_000337795.1	3.82597	ND	55	[40]
Haloferacaceae	*Haloferax elongans*	ATCC BAA-1513	GCA_000336755.1	3.95214	53	55	[41]
Haloferacaceae	*Haloferax gibbonsii*	ATCC 33959	GCA_000336775.1	4.05732	40	55	[42]
Haloferacaceae	*Haloferax larsenii*	JCM 13917	GCA_000336955.1	3.69795	45	55	[43]
Haloferacaceae	*Haloferax lucentense*	DSM 14919	GCA_000336795.1	3.61906	37	45	[44]
Haloferacaceae	*Haloferax mediterranei*	ATCC 33500	GCA_000685635.1	3.90670	51	55	[45]
Haloferacaceae	*Haloferax mucosum*	ATCC BAA-1512	GCA_000337815.1	3.36898	53	55	[41]
Haloferacaceae	*Haloferax prahovense*	DSM 18310	GCA_000336815.1	3.99880	48	51	[46]
Haloferacaceae	*Haloferax volcanii*	DS2	GCA_000025685.1	4.01290	45	49	[13]
Haloferacaceae	*Halogeometricum borinquense*	PR 3	GCA_000172995.2	3.94447	40	50	[47]
Haloferacaceae	*Halogeometricum limi*	CGMCC 1.8711	GCA_900115785.1	3.61627	37	50	[48,49]
Haloferacaceae	*Halogeometricum pallidum*	JCM 14848	GCA_000337095.1	4.38452	30	45	[48,50]
Haloferacaceae	*Halogeometricum rufum*	CGMCC 1.7736	GCA_900112175.1	4.18712	42	55	[51]
Haloferacaceae	*Halopelagius inordinatus*	CGMCC 1.7739	GCA_900113245.1	3.52931	37	50	[52]

Haloferacaceae	*Haloplanus natans*	DSM 17983	GCA_000427685.1	3.79793	40	52	[53]
Haloferacaceae	*Haloplanus vescus*	CGMCC 1.8712	GCA_900107665.1	2.77686	40	50	[52]
Haloferacaceae	*Haloprofundus marisrubri*	SB9	GCA_001469955.1	3.92956	35	45	[54]
Haloferacaceae	*Haloquadratum walsbyi*	DSM 16854	GCA_000237865.1	3.26048	45	55	[55]
Halorubraceae	*Halobaculum gomorrense*	DSM 9297	GCA_900129775.1	3.20825	40	45	[56]
Halorubraceae	*Halogranum amylolyticum*	CGMCC 1.10121	GCA_900110465.1	5.18569	37	50	[57]
Halorubraceae	*Halogranum gelatinilyticum*	CGMCC 1.10119	GCA_900103715.1	3.77019	37	50	[57]
Halorubraceae	*Halogranum rubrum*	CGMCC 1.7738	GCA_900114455.1	4.56668	37	50	[58]
Halorubraceae	*Halogranum salarium*	B-1	GCA_000283335.1	4.49231	37	50	[59]
Halorubraceae	*Halohasta litchfieldiae*	DSM 22187	GCA_900109065.1	3.28459	30	45	[60]
Halorubraceae	*Halolamina pelagica*	CGMCC 1.10329	GCA_900115675.1	3.16063	37	50	[61]
Halorubraceae	*Halolamina rubra*	CBA1107	GCA_000739555.1	2.95500	37	50	[62]
Halorubraceae	*Halolamina sediminis*	Halo7	GCA_001282785.1	2.83586	40	45	[63]
Halorubraceae	*Halopenitus malekzadehii*	IBRC-M 10418	GCA_900108505.1	3.13682	40	50	[64]
Halorubraceae	*Halopenitus persicus*	KCTC 4046	GCA_900107205.1	3.42449	40	50	[65]
Halorubraceae	*Halorubrum aidingense*	JCM 13560	GCA_000336995.1	3.10853	42	52	[66]
Halorubraceae	*Halorubrum arcis*	JCM 13916	GCA_000337015.1	3.38260	42	55	[67]
Halorubraceae	*Halorubrum californiense*	DSM 19288	GCA_000336875.1	3.68287	37	42	[68]
Halorubraceae	*Halorubrum coriense*	DSM 10284	GCA_000337035.1	3.64531	50	56	[69]
Halorubraceae	*Halorubrum distributum*	JCM 10118	GCA_000337335.1	3.30613	45	50	[69]
Halorubraceae	*Halorubrum ezzemoulense*	DSM 17463	GCA_000421805.1	3.59148	40	50	[70]
Halorubraceae	*Halorubrum halophilum*	B8	GCA_000739595.1	3.67798	40	45	[71]
Halorubraceae	*Halorubrum kocurii*	JCM 14978	GCA_000337355.1	3.61974	37	55	[72]
Halorubraceae	*Halorubrum lacusprofundi*	ATCC 49239	GCA_000022205.1	3.69258	37	45	[73]
Halorubraceae	*Halorubrum lipolyticum*	DSM 21995	GCA_000337375.1	3.42504	48	58	[66]
Halorubraceae	*Halorubrum litoreum*	JCM 13561	GCA_000337395.1	3.13776	42	55	[74]
Halorubraceae	*Halorubrum saccharovorum*	DSM 1137	GCA_000337915.1	3.42370	50	56	[75]

Halorubraceae	*Halorubrum sodomense*	RD 26	GCA_900111935.1	3.03055	40	50	[75]
Halorubraceae	*Halorubrum tebenquichense*	DSM 14210	GCA_000337415.1	3.32886	ND	50	[76]
Halorubraceae	*Halorubrum terrestre*	JCM 10247	GCA_000337435.1	3.37622	45	50	[77]
Natrialbaceae	*Halobiforma haloterrestris*	DSM 13078	GCA_900112205.1	4.49544	42	58	[78]
Natrialbaceae	*Halobiforma lacisalsi*	AJ5	GCA_000226975.3	4.38154	45	57	[79]
Natrialbaceae	*Halobiforma nitratireducens*	JCM 10879	GCA_000337895.1	3.68875	41	44	[78,80]
Natrialbaceae	*Halopiger salifodinae*	CGMCC 1.12284	GCA_900110455.1	4.27217	45	50	[81]
Natrialbaceae	*Halopiger xanaduensis*	SH-6	GCA_000217715.1	4.35527	37	45	[82]
Natrialbaceae	*Halostagnicola kamekurae*	DSM 22427	GCA_900116205.1	4.10815	30	45	[83]
Natrialbaceae	*Halostagnicola larsenii*	XH-48	GCA_000517625.1	4.13118	37	50	[84]
Natrialbaceae	*Haloterrigena daqingensis*	JX313	GCA_001971705.1	3.83580	35	50	[85]
Natrialbaceae	*Haloterrigena jeotgali*	A29	GCA_000710605.1	4.13162	45	50	[86]
Natrialbaceae	*Haloterrigena limicola*	JCM 13563	GCA_000337475.1	3.52203	50	61	[87]
Natrialbaceae	*Haloterrigena saccharevitans*	AB14	GCA_001953745.1	3.98062	45	58	[88]
Natrialbaceae	*Haloterrigena salina*	JCM 13891	GCA_000337495.1	4.84161	37	50	[89]
Natrialbaceae	*Haloterrigena thermotolerans*	DSM 11522	GCA_000337115.1	3.89527	50	60	[90]
Natrialbaceae	*Haloterrigena turkmenica*	DSM 5511	GCA_000025325.1	5.44078	45	57	[91]; [13]
Natrialbaceae	*Halovivax asiaticus*	JCM 14624	GCA_000337515.1	3.23845	37	50	[84]
Natrialbaceae	*Halovivax ruber*	XH-70	GCA_000328525.1	3.22388	37	45	[92]
Natrialbaceae	*Natrialba aegyptia*	DSM 13077	GCA_000337535.1	4.61836	42	60	[93]
Natrialbaceae	*Natrialba asiatica*	DSM 12278	GCA_000337555.1	4.40418	40	50	[93]
Natrialbaceae	*Natrialba chahannaoensis*	JCM 10990	GCA_000337135.1	4.30927	45	55	[94]
Natrialbaceae	*Natrialba hulunbeirensis*	JCM 10989	GCA_000337575.1	4.15961	50	55	[94]
Natrialbaceae	*Natrialba magadii*	ATCC 43099	GCA_000025625.1	4.44364	40	50	[95]
Natrialbaceae	*Natrialba taiwanensis*	DSM 12281	GCA_000337595.1	4.63519	40	55	[93]
Natrialbaceae	*Natrinema altunense*	AJ2	GCA_000731985.1	3.77413	ND	ND	
Natrialbaceae	*Natrinema ejinorense*	JCM 13890	NXNI00000000	4.47632	37	50	[6]

Natrialbaceae	*Natrinema gari*	JCM 14663	GCA_000337175.1	4.02369	40	60	[96]
Natrialbaceae	*Natrinema pallidum*	DSM 3751	GCA_000337615.1	3.91581	40	ND	[97]
Natrialbaceae	*Natrinema pellirubrum*	DSM 15624	GCA_000230735.3	4.35410	40	45	[97]
Natrialbaceae	*Natrinema salaciae*	DSM 25055	GCA_900110865.1	4.85702	45	52.5	[2]
Natrialbaceae	*Natrinema thermophila*	CBA1119	PDBS00000000	5.05806	55	66	From this study
Natrialbaceae	*Natrinema versiforme*	JCM 10478	GCA_000337195.1	4.19080	46	53	[98]
Natrialbaceae	*Natronobacterium gregoryi*	SP2	GCA_000230715.3	3.78836	37	40	[95]
Natrialbaceae	*Natronobacterium texcoconense*	DSM 24767	GCA_900104065.1	4.00987	37	45	[99]
Natrialbaceae	*Natronococcus amylolyticus*	DSM 10524	GCA_000337675.1	4.41653	45	50	[100]
Natrialbaceae	*Natronococcus jeotgali*	DSM 18795	GCA_000337695.1	4.49618	45	50	[101]
Natrialbaceae	*Natronococcus occultus*	SP4	GCA_000328685.1	4.31412	45	54	[13]
Natrialbaceae	*Natronolimnobius baerhuensis*	JCM 12253	GCA_001316185.1	3.89306	37	46	[102]
Natrialbaceae	*Natronolimnobius innermongolicus*	JCM 12255	GCA_000337215.1	4.58863	45	54	[102]
Natrialbaceae	*Natronorubrum bangense*	JCM 10635	GCA_000337715.1	4.11128	45	55	[103]
Natrialbaceae	*Natronorubrum sediminis*	CGMCC 1.8981	GCA_900108095.1	3.78254	37	50	[104]
Natrialbaceae	*Natronorubrum sulfidifaciens*	JCM 14089	GCA_000337735.1	3.46029	47	55	[105]
Natrialbaceae	*Natronorubrum texcoconense*	DSM 25211	GCA_900100335.1	4.64179	37	45	[106]
Natrialbaceae	*Natronorubrum tibetense*	GA33	GCA_000383975.1	4.93484	45	ND	[103]

*ND, No data

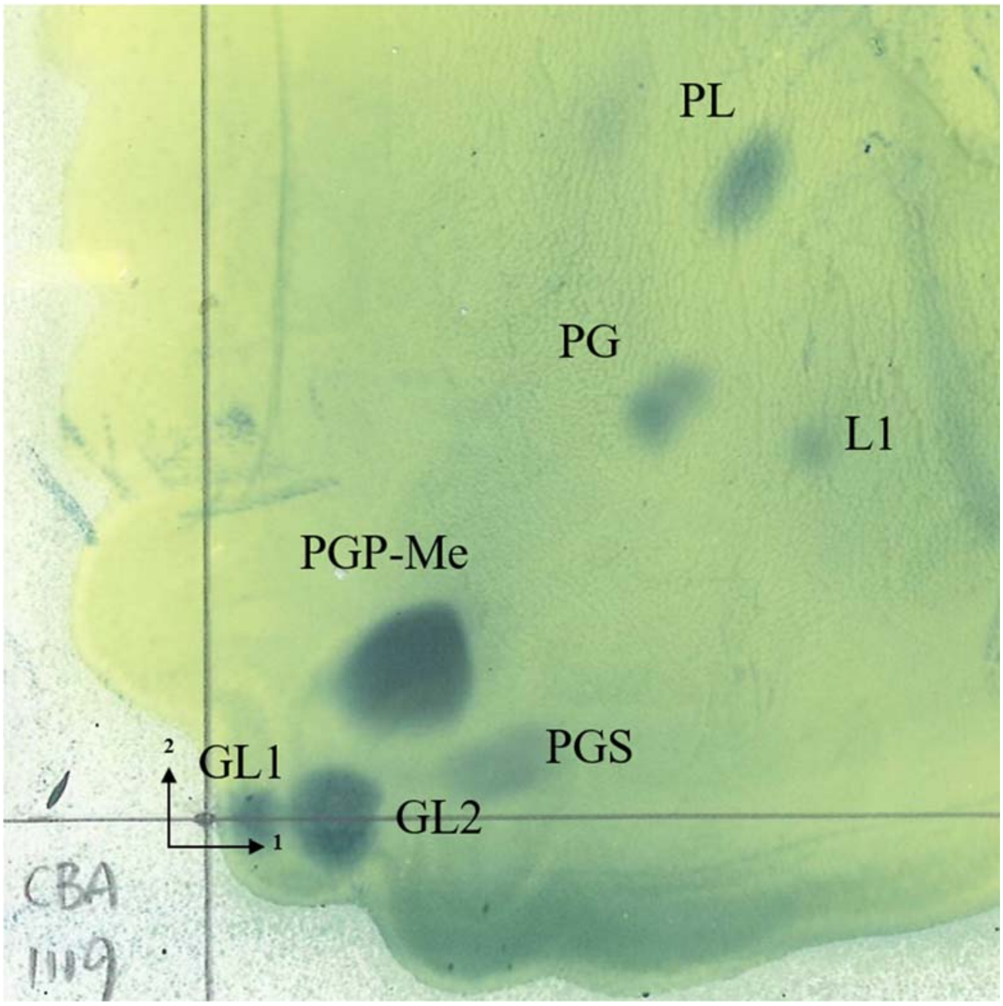

Supplementary Figure S1. High performance thin-layer chromatogram of total lipids from strain CBA1119^T. Total lipids were detected using 5% ethanolic molybdophosphoric acid. Abbreviations: PG, phosphatidylglycerol; PGP-Me, phosphatidylglycerol phosphate methyl ester; PGS, phosphatidylglycerol sulfate; GL1–2, unidentified glycolipids; PL, unidentified phospholipid; L1, unidentified lipid.

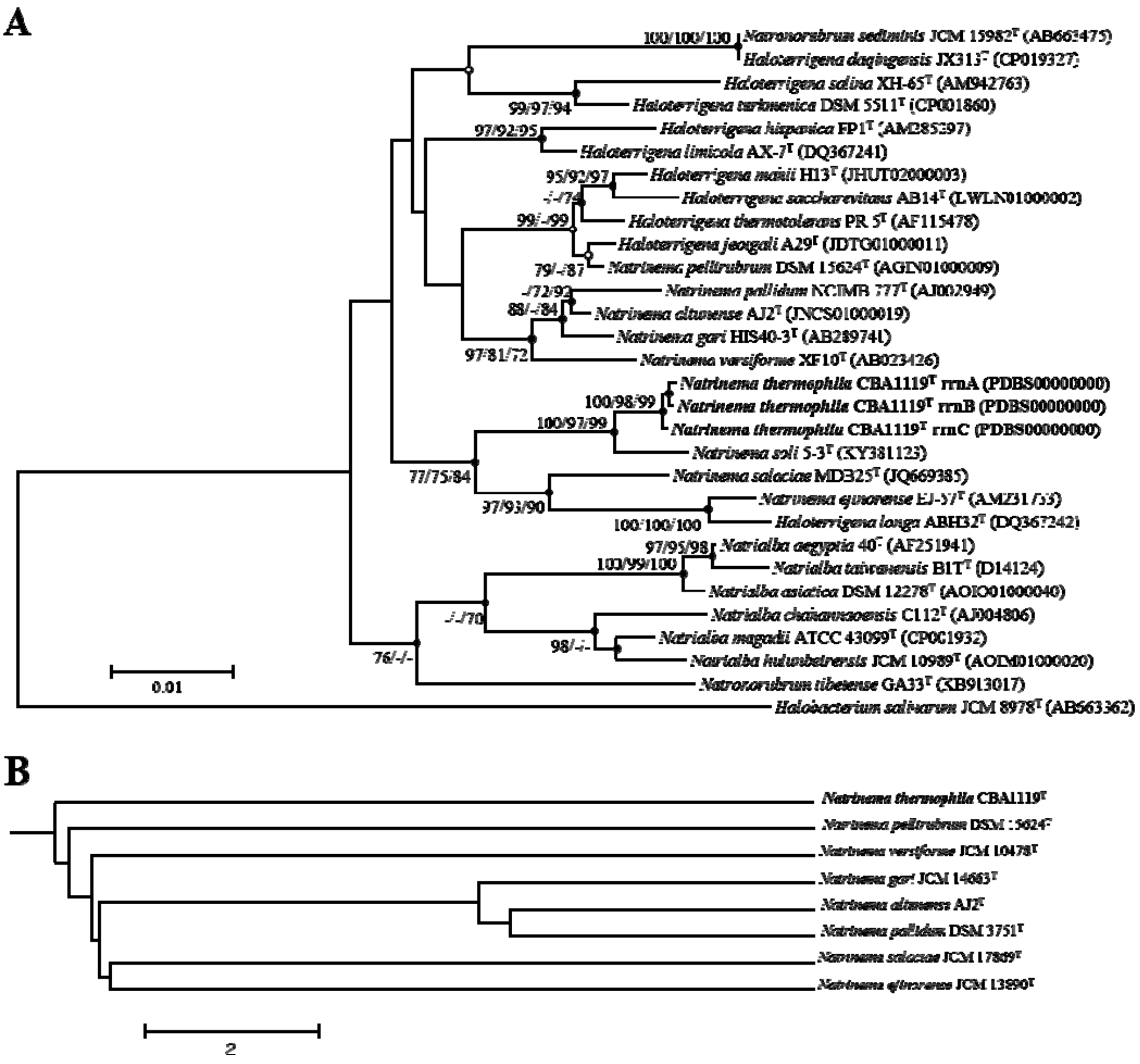

Supplementary Figure S2. Phylogenetic trees. (A) Tree based on 16S rRNA gene sequences of strain CBA1119^{T} and related species. The tree was constructed based on the neighbor-joining (NJ) algorithm. Bootstrap values (> 70%) at the nodes were calculated using probabilities of the NJ, maximum likelihood (ML), and maximum parsimony (MP) algorithms. Closed circles indicate nodes determined with both ML and MP algorithms, and open circles indicate nodes determined with ML or MP algorithm. *Halobacterium salinarum* JCM 8978^{T} was used as the outgroup species. (B) Tree based on OrthoANI values of strain CBA1119^{T} and other *Natrinema* spp.

REFERENCES

1. Wayne, L. G. *et al.* Report of the Ad Hoc Committee on Reconciliation of Approaches to Bacterial Systematics. *Int J Syst Evol Microbiol* **37**, 463-464 (1987).
2. Albuquerque, L., Taborda, M., La Cono, V., Yakimov, M. & da Costa, M. S. *Natrinema salaciae* sp. nov., a halophilic archaeon isolated from the deep, hypersaline anoxic Lake Medee in the Eastern Mediterranean Sea. *Syst Appl Microbiol* **35**, 368-373 (2012).
3. Minegishi, H., Kamekura, M., Itoh, T., Echigo, A., Usami, R. & Hashimoto, T. Further refinement of the phylogeny of the Halobacteriaceae based on the full-length RNA polymerase subunit B' (*rpoB'*) gene. *Int J Syst Evol Microbiol* **60**, 2398-2408 (2010).
4. Rasooli, M. *et al. Natrinema soli* sp. nov., a novel halophilic archaeon isolated from a hypersaline wetland. *Int J Syst Evol Microbiol* **67**, 2142-2147 (2017).
5. Tindall, B. J. Taxonomic problems arising in the genera *Haloterrigena* and *Natrinema*. *Int J Syst Evol Microbiol* **53**, 1697-1698 (2003).
6. Castillo, A. M. *et al. Natrinema ejinorense* sp. nov., isolated from a saline lake in Inner Mongolia, China. *Int J Syst Evol Microbiol* **56**, 2683-2687 (2006).
7. Song, H. S. *et al. Halapricum salinum* gen. nov., sp. nov., an extremely halophilic archaeon isolated from non-purified solar salt. *Antonie Van Leeuwenhoek* **105**, 979-986 (2014).
8. Yang, Y., Cui, H. L., Zhou, P. J. & Liu, S. J. *Haloarcula amylolytica* sp. nov., an extremely halophilic archaeon isolated from Aibi salt lake in Xin-Jiang, China. *Int J Syst Evol Microbiol* **57**, 103-106 (2007).
9. Ihara, K., Watanabe, S. & Tamura, T. *Haloarcula argentinensis* sp. nov. and *Haloarcula mukohataei* sp. nov., two new extremely halophilic archaea collected in Argentina. *Int J Syst Bacteriol* **47**, 73-77 (1997).
10. Ventosa, A. *Haloarcula*. In: *Bergey's Manual of Systematics of Archaea and Bacteria*.

John Wiley & Sons, Ltd (2015).

11. Takashina, T., Hamamoto, T., Otozai, K., Grant, W. D. & Horikoshi, K. *Haloarcula japonica* sp. nov., a new triangular halophilic archaebacterium. *Syst Appl Microbiol* **13**, 177-181 (1990).

12. Oren, A., Ginzburg, M., Ginzburg, B. Z., Hochstein, L. I. & Volcani, B. E. *Haloarcula marismortui* (Volcani) sp. nov., nom. rev., an extremely halophilic bacterium from the Dead Sea. *Int J Syst Bacteriol* **40**, 209-210 (1990).

13. Robinson, J. L. *et al.* Growth Kinetics of Extremely Halophilic Archaea (Family Halobacteriaceae) as Revealed by Arrhenius Plots. *J Bacteriol* **187**, 923-929 (2005).

14. Kharroub, K. *et al. Halomicrobium katesii* sp. nov., an extremely halophilic archaeon. *Int J Syst Evol Microbiol* **58**, 2354-2358 (2008).

15. Oren, A., Elevi, R., Watanabe, S., Ihara, K. & Corcelli, A. *Halomicrobium mukohataei* gen. nov., comb. nov., and emended description of *Halomicrobium mukohataei*. *Int J Syst Evol Microbiol* **52**, 1831-1835 (2002).

16. Yang, X. & Cui, H. L. *Halomicrobium zhouii* sp. nov., a halophilic archaeon from a marine solar saltern. *Int J Syst Evol Microbiol* **62**, 1235-1240 (2012).

17. Antunes, A., Taborda, M., Huber, R., Moissl, C., Nobre, M. F. & da Costa, M. S. *Halorhabdus tiamatea* sp. nov., a non-pigmented, extremely halophilic archaeon from a deep-sea, hypersaline anoxic basin of the Red Sea, and emended description of the genus *Halorhabdus*. *Int J Syst Evol Microbiol* **58**, 215-220 (2008).

18. Waino, M., Tindall, B. J. & Ingvorsen, K. *Halorhabdus utahensis* gen. nov., sp. nov., an aerobic, extremely halophilic member of the Archaea from Great Salt Lake, Utah. *Int J Syst Evol Microbiol*, 183-190 (2000).

19. Amoozegar, M. A., Makhdoumi-Kakhki, A., Mehrshad, M., Fazeli, S. A., Sproer, C. & Ventosa, A. *Halorientalis persicus* sp. nov., an extremely halophilic archaeon isolated

from a salt lake and emended description of the genus *Halorientalis*. *Int J Syst Evol Microbiol* **64**, 940-944 (2014).

20. Vreeland, R. H., Straight, S., Krammes, J., Dougherty, K., Rosenzweig, W. D. & Kamekura, M. *Halosimplex carlsbadense* gen. nov., sp. nov., a unique halophilic archaeon, with three 16S rRNA genes, that grows only in defined medium with glycerol and acetate or pyruvate. *Extremophiles* **6**, 445-452 (2002).

21. Roh, S. W., Lee, M. L. & Bae, J. W. *Haladaptatus cibarius* sp. nov., an extremely halophilic archaeon from seafood, and emended description of the genus *Haladaptatus*. *Int J Syst Evol Microbiol* **60**, 1187-1190 (2010).

22. Cui, H. L. *et al. Haladaptatus litoreus* sp. nov., an extremely halophilic archaeon from a marine solar saltern, and emended description of the genus *Haladaptatus*. *Int J Syst Evol Microbiol* **60**, 1085-1089 (2010).

23. Savage, K. N., Krumholz, L. R., Oren, A. & Elshahed, M. S. *Haladaptatus paucihalophilus* gen. nov., sp. nov., a halophilic archaeon isolated from a low-salt, sulfide-rich spring. *Int J Syst Evol Microbiol* **57**, 19-24 (2007).

24. Roh, S. W. *et al. Halalkalicoccus jeotgali* sp. nov., a halophilic archaeon from shrimp jeotgal, a traditional Korean fermented seafood. *Int J Syst Evol Microbiol* **57**, 2296-2298 (2007).

25. Liu, B. B. *et al. Halalkalicoccus paucihalophilus* sp. nov., a halophilic archaeon from Lop Nur region in Xinjiang, northwest of China. *Antonie Van Leeuwenhoek* **103**, 1007-1014 (2013).

26. Minegishi, H., Echigo, A., Nagaoka, S., Kamekura, M. & Usami, R. *Halarchaeum acidiphilum* gen. nov., sp. nov., a moderately acidophilic haloarchaeon isolated from commercial solar salt. *Int J Syst Evol Microbiol* **60**, 2513-2516 (2010).

27. Makhdoumi-Kakhki, A., Amoozegar, M. A., Bagheri, M., Ramezani, M. & Ventosa, A.

Haloarchaeobius iranensis gen. nov., sp. nov., an extremely halophilic archaeon isolated from a saline lake. *Int J Syst Evol Microbiol* **62**, 1021-1026 (2012).

28. Yang, Y., Cui, H. L., Zhou, P. J. & Liu, S. J. *Halobacterium jilantaiense* sp. nov., a halophilic archaeon isolated from a saline lake in Inner Mongolia, China. *Int J Syst Evol Microbiol* **56**, 2353-2355 (2006).
29. Sorokin, D. Y. *et al.* Discovery of anaerobic lithoheterotrophic haloarchaea, ubiquitous in hypersaline habitats. *ISME J* **11**, 1245-1260 (2017).
30. Makhdoumi-Kakhki, A., Amoozegar, M. A. & Ventosa, A. *Halovenus aranensis* gen. nov., sp. nov., an extremely halophilic archaeon from Aran-Bidgol salt lake. *Int J Syst Evol Microbiol* **62**, 1331-1336 (2012).
31. Burns, D. G. *et al. Natronomonas moolapensis* sp. nov., non-alkaliphilic isolates recovered from a solar saltern crystallizer pond, and emended description of the genus *Natronomonas*. *Int J Syst Evol Microbiol* **60**, 1173-1176 (2010).
32. Goh, F. *et al. Halococcus hamelinensis* sp. nov., a novel halophilic archaeon isolated from stromatolites in Shark Bay, Australia. *Int J Syst Evol Microbiol* **56**, 1323-1329 (2006).
33. Montero, C. G., Ventosa, A., Rodriguez-Valera, F., Kates, M., Moldoveanu, N. & Ruiz-Berraquero, F. *Halococcus saccharolyticus* sp. nov., a new species of extremely halophilic non-alkaliphilic cocci. *Syst Appl Microbiol* **12**, 167-171 (1989).
34. Denner, E. B., McGENITY, T. J., BUSSE, H.-J., Grant, W. D., Wanner, G. & Stan-Lotter, H. *Halococcus salifodinae* sp. nov., an archaeal isolate from an Austrian salt mine. *Int J Syst Evol Microbiol* **44**, 774-780 (1994).
35. Yim, K. J. *et al. Halococcus sediminicola* sp. nov., an extremely halophilic archaeon isolated from a marine sediment. *Antonie Van Leeuwenhoek* **105**, 73-79 (2014).
36. Namwong, S., Tanasupawat, S., Visessanguan, W., Kudo, T. & Itoh, T. *Halococcus*

thailandensis sp. nov., from fish sauce in Thailand. *Int J Syst Evol Microbiol* **57**, 2199-2203 (2007).

37. Cui, H. L., Yang, X., Gao, X. & Xu, X. W. *Halobellus clavatus* gen. nov., sp. nov. and *Halorientalis regularis* gen. nov., sp. nov., two new members of the family *Halobacteriaceae*. *Int J Syst Evol Microbiol* **61**, 2682-2689 (2011).
38. Cha, I. T. *et al. Halobellus rufus* sp. nov., an extremely halophilic archaeon isolated from non-purified solar salt. *Antonie Van Leeuwenhoek* **105**, 925-932 (2014).
39. Asker, D. & Ohta, Y. *Haloferax alexandrinus* sp. nov., an extremely halophilic canthaxanthin-producing archaeon from a solar saltern in Alexandria (Egypt). *Int J Syst Evol Microbiol* **52**, 729-738 (2002).
40. Tindall, B. J., Tomlinson, G. A. & Hochstein, L. I. Transfer of *Halobacterium denitrificans* (Tomlinson, Jahnke, and Hochstein) to the genus *Haloferax* as *Haloferax denitrificans* comb. nov. *Int J Syst Bacteriol* **39**, 359-360 (1989).
41. Allen, M. A. *et al. Haloferax elongans* sp. nov. and *Haloferax mucosum* sp. nov., isolated from microbial mats from Hamelin Pool, Shark Bay, Australia. *Int J Syst Evol Microbiol* **58**, 798-802 (2008).
42. Juez, G., Rodriguez-Valera, F., Ventosa, A. & Kushner, D. J. *Haloarcula hispanica* spec. nov. and *Haloferax gibbonsii* spec, nov., two new species of extremely halophilic archaebacteria. *Syst Appl Microbiol* **8**, 75-79 (1986).
43. Xu, X. W., Wu, Y. H., Wang, C. S., Oren, A., Zhou, P. J. & Wu, M. *Haloferax larsenii* sp. nov., an extremely halophilic archaeon from a solar saltern. *Int J Syst Evol Microbiol* **57**, 717-720 (2007).
44. Gutierrez, M. C., Kamekura, M., Holmes, M. L., Dyall-Smith, M. L. & Ventosa, A. Taxonomic characterization of *Haloferax* sp. (" *H. alicantei*") strain Aa 2.2: description of *Haloferax lucentensis* sp. nov. *Extremophiles* **6**, 479-483 (2002).

45. Ventosa, A. *Haloferax*. In: *Bergey's Manual of Systematics of Archaea and Bacteria*. John Wiley & Sons, Ltd (2015).

46. Enache, M., Itoh, T., Kamekura, M., Teodosiu, G. & Dumitru, L. *Haloferax prahovense* sp. nov., an extremely halophilic archaeon isolated from a Romanian salt lake. *Int J Syst Evol Microbiol* **57**, 393-397 (2007).

47. Montalvo-Rodriguez, R., Vreeland, R. H., Oren, A., Kessel, M., Betancourt, C. & Lopez-Garriga, J. *Halogeometricum borinquense* gen. nov., sp. nov., a novel halophilic archaeon from Puerto Rico. *Int J Syst Bacteriol*, 1305-1312 (1998).

48. Qiu, X. X., Zhao, M. L., Han, D., Zhang, W. J., Dyall-Smith, M. L. & Cui, H. L. Taxonomic study of the genera *Halogeometricum* and *Halosarcina*: transfer of *Halosarcina limi* and *Halosarcina pallida* to the genus *Halogeometricum* as *Halogeometricum limi* comb. nov. and *Halogeometricum pallidum* comb. nov., respectively. *Int J Syst Evol Microbiol* **63**, 3915-3919 (2013).

49. Cui, H. L. *et al. Halosarcina limi* sp. nov., a halophilic archaeon from a marine solar saltern, and emended description of the genus *Halosarcina*. *Int J Syst Evol Microbiol* **60**, 2462-3466 (2010).

50. Savage, K. N., Krumholz, L. R., Oren, A. & Elshahed, M. S. *Halosarcina pallida* gen. nov., sp. nov., a halophilic archaeon from a low-salt, sulfide-rich spring. *Int J Syst Evol Microbiol* **58**, 856-860 (2008).

51. Cui, H. L. *et al. Halogeometricum rufum* sp. nov., a halophilic archaeon from a marine solar saltern, and emended description of the genus *Halogeometricum*. *Int J Syst Evol Microbiol* **60**, 2613-2617 (2010).

52. Cui, H. L. *et al. Halopelagius inordinatus* gen. nov., sp. nov., a new member of the family *Halobacteriaceae* isolated from a marine solar saltern. *Int J Syst Evol Microbiol* **60**, 2089-2093 (2010).

53. Bardavid, R. E., Mana, L. & Oren, A. *Haloplanus natans* gen. nov., sp. nov., an extremely halophilic, gas-vacuolate archaeon isolated from Dead Sea-Red Sea water mixtures in experimental outdoor ponds. *Int J Syst Evol Microbiol* **57**, 780-783 (2007).

54. Zhang, G. *et al. Haloprofundus marisrubri* gen. nov., sp. nov., an extremely halophilic archaeon isolated from a brine–seawater interface. *Int J Syst Evol Microbiol* **67**, 9-16 (2017).

55. Burns, D. G. *et al. Haloquadratum walsbyi* gen. nov., sp. nov., the square haloarchaeon of Walsby, isolated from saltern crystallizers in Australia and Spain. *Int J Syst Evol Microbiol* **57**, 387-392 (2007).

56. Oren, A., Gurevich, P., Gemmell, R. T. & Teske, A. *Halobaculum gomorrense* gen. nov., sp. nov., a novel extremely halophilic archaeon from the Dead Sea. *Int J Syst Bacteriol* **45**, 747-754 (1995).

57. Cui, H. L., Yang, X., Gao, X. & Xu, X. W. *Halogranum gelatinilyticum* sp. nov. and *Halogranum amylolyticum* sp. nov., isolated from a marine solar saltern, and emended description of the genus *Halogranum*. *Int J Syst Evol Microbiol* **61**, 911-915 (2011).

58. Cui, H. L. *et al. Haloplanus vescus* sp. nov., an extremely halophilic archaeon from a marine solar saltern, and emended description of the genus *Haloplanus*. *Int J Syst Evol Microbiol* **60**, 1824-1827 (2010).

59. Kim, K. K., Lee, K. C. & Lee, J. S. *Halogranum salarium* sp. nov., a halophilic archaeon isolated from sea salt. *Syst Appl Microbiol* **34**, 576-580 (2011).

60. Mou, Y. Z., Qiu, X. X., Zhao, M. L., Cui, H. L., Oh, D. & Dyall-Smith, M. L. *Halohasta litorea* gen. nov. sp. nov., and *Halohasta litchfieldiae* sp. nov., isolated from the Daliang aquaculture farm, China and from Deep Lake, Antarctica, respectively. *Extremophiles* **16**, 895-901 (2012).

61. Cui, H. L., Gao, X., Yang, X. & Xu, X. W. *Halolamina pelagica* gen. nov., sp. nov., a

new member of the family *Halobacteriaceae*. *Int J Syst Evol Microbiol* **61**, 1617-1621 (2011).

62. Cha, I. T. *et al. Halolamina rubra* sp. nov., a haloarchaeon isolated from non-purified solar salt. *Antonie Van Leeuwenhoek* **105**, 907-914 (2014).

63. Koh, H. W., Song, H. S., Song, U., Yim, K. J., Roh, S. W. & Park, S. J. *Halolamina sediminis* sp. nov., an extremely halophilic archaeon isolated from solar salt. *Int J Syst Evol Microbiol* **65**, 2479-2484 (2015).

64. Amoozegar, M. A., Makhdoumi-Kakhki, A., Mehrshad, M., Fazeli, S. A. & Ventosa, A. *Halopenitus malekzadehii* sp. nov., an extremely halophilic archaeon isolated from a salt lake. *Int J Syst Evol Microbiol* **63**, 3232-3236 (2013).

65. Amoozegar, M. A., Makhdoumi-Kakhki, A., Shahzadeh Fazeli, S. A., Azarbaijani, R. & Ventosa, A. *Halopenitus persicus* gen. nov., sp. nov., an archaeon from an inland salt lake. *Int J Syst Evol Microbiol* **62**, 1932-1936 (2012).

66. Cui, H. L., Tohty, D., Zhou, P. J. & Liu, S. J. *Halorubrum lipolyticum* sp. nov. and *Halorubrum aidingense* sp. nov., isolated from two salt lakes in Xin-Jiang, China. *Int J Syst Evol Microbiol* **56**, 1631-1634 (2006).

67. Xu, X. W., Wu, Y. H., Zhang, H. B. & Wu, M. *Halorubrum arcis* sp. nov., an extremely halophilic archaeon isolated from a saline lake on the Qinghai-Tibet Plateau, China. *Int J Syst Evol Microbiol* **57**, 1069-1072 (2007).

68. Pesenti, P. T., Sikaroodi, M., Guillevet, P. M., Sanchez-Porro, C., Ventosa, A. & Litchfield, C. D. *Halorubrum californiense* sp. nov., an extreme archaeal halophile isolated from a crystallizer pond at a solar salt plant in California. *Int J Syst Evol Microbiol* **58**, 2710-2715 (2008).

69. McGenity, T. J. & Grant, W. D. *Halorubrum*. In: *Bergey's Manual of Systematics of Archaea and Bacteria*. John Wiley & Sons, Ltd (2015).

70. Kharroub, K. *et al. Halorubrum ezzemoulense* sp. nov., a halophilic archaeon isolated from Ezzemoul sabkha, Algeria. *Int J Syst Evol Microbiol* **56**, 1583-1588 (2006).

71. Yim, K. J. *et al. Halorubrum halophilum* sp. nov., an extremely halophilic archaeon isolated from a salt-fermented seafood. *Antonie Van Leeuwenhoek* **105**, 603-612 (2014).

72. Gutiérrez, M. C. *et al. Halorubrum kocurii* sp. nov., an archaeon isolated from a saline lake. *Int J Syst Evol Microbiol* **58**, 2031-2035 (2008).

73. Franzmann, P. D. *et al. Halobacterium lacusprofundi* sp. nov., a Halophilic Bacterium Isolated from Deep Lake, Antarctica. *Syst Appl Microbiol* **11**, 20-27 (1988).

74. Cui, H. L., Lin, Z. Y., Dong, Y., Zhou, P. J. & Liu, S. J. *Halorubrum litoreum* sp. nov., an extremely halophilic archaeon from a solar saltern. *Int J Syst Evol Microbiol* **57**, 2204-2206 (2007).

75. McGenity, T. J. & Grant, W. D. Transfer of *Halobacterium saccharovorum*, *Halobacterium sodomense*, *Halobacterium trapanicum* NRC 34021 and *Halobacterium lacusprofundi* to the genus *Halorubrum* gen. nov., as *Halorubrum saccharovorum* comb. nov., *Halorubrum sodomense* comb. nov., *Halorubrum trapanicum* comb. nov., and *Halorubrum lacusprofundi* comb. nov. *Syst Appl Microbiol* **18**, 237-243 (1995).

76. Lizama, C. *et al. Halorubrum tebenquichense* sp. nov., a novel halophilic archaeon isolated from the Atacama Saltern, Chile. *Int J Syst Evol Microbiol* **52**, 149-155 (2002).

77. Fan, H., Xue, Y., Ma, Y., Ventosa, A. & Grant, W. D. *Halorubrum tibetense* sp. nov., a novel haloalkaliphilic archaeon from Lake Zabuye in Tibet, China. *Int J Syst Evol Microbiol* **54**, 1213-1216 (2004).

78. Hezayen, F. F., Tindall, B. J., Steinbuchel, A. & Rehm, B. H. Characterization of a novel halophilic archaeon, *Halobiforma haloterrestris* gen. nov., sp. nov., and transfer of *Natronobacterium nitratireducens* to *Halobiforma nitratireducens* comb. nov. *Int J Syst*

Evol Microbiol **52**, 2271-2280 (2002).

79. Xu, X. W., Wu, M., Zhou, P. J. & Liu, S. J. *Halobiforma lacisalsi* sp. nov., isolated from a salt lake in China. *Int J Syst Evol Microbiol* **55**, 1949-1952 (2005).

80. Xin, H., Itoh, T., Zhou, P., Suzuki, K. & Nakase, T. *Natronobacterium nitratireducens* sp. nov., a haloalkaliphilic archaeon isolated from a soda lake in China. *Int J Syst Evol Microbiol* **51**, 1825-1829 (2001).

81. Zhang, W. Y., Meng, Y., Zhu, X. F. & Wu, M. *Halopiger salifodinae* sp. nov., an extremely halophilic archaeon isolated from a salt mine. *Int J Syst Evol Microbiol* **63**, 3563-3567 (2013).

82. Gutiérrez, M. C. *et al. Halopiger xanaduensis* gen. nov., sp. nov., an extremely halophilic archaeon isolated from saline Lake Shangmatala in Inner Mongolia, China. *Int J Syst Evol Microbiol* **57**, 1402-1407 (2007).

83. Nagaoka, S., Minegishi, H., Echigo, A. & Usami, R. *Halostagnicola kamekurae* sp. nov., an extremely halophilic archaeon from solar salt. *Int J Syst Evol Microbiol* **60**, 2828-2831 (2010).

84. Castillo, A. M. *et al. Halostagnicola larsenii* gen. nov., sp. nov., an extremely halophilic archaeon from a saline lake in Inner Mongolia, China. *Int J Syst Evol Microbiol* **56**, 1519-1524 (2006).

85. Wang, S. *et al. Haloterrigena daqingensis* sp. nov., an extremely haloalkaliphilic archaeon isolated from a saline-alkaline soil. *Int J Syst Evol Microbiol* **60**, 2267-2271 (2010).

86. Roh, S. W. *et al. Haloterrigena jeotgali* sp. nov., an extremely halophilic archaeon from salt-fermented food. *Int J Syst Evol Microbiol* **59**, 2359-2363 (2009).

87. Cui, H. L., Tohty, D., Zhou, P. J. & Liu, S. J. *Haloterrigena longa* sp. nov. and *Haloterrigena limicola* sp. nov., extremely halophilic archaea isolated from a salt lake.

Int J Syst Evol Microbiol **56**, 1837-1840 (2006).

88. Xu, X. W., Liu, S. J., Tohty, D., Oren, A., Wu, M. & Zhou, P. J. *Haloterrigena saccharevitans* sp. nov., an extremely halophilic archaeon from Xin-Jiang, China. *Int J Syst Evol Microbiol* **55**, 2539-2542 (2005).
89. Gutierrez, M. C., Castillo, A. M., Kamekura, M. & Ventosa, A. *Haloterrigena salina* sp. nov., an extremely halophilic archaeon isolated from a salt lake. *Int J Syst Evol Microbiol* **58**, 2880-2884 (2008).
90. Montalvo-Rodriguez, R., Lopez-Garriga, J., Vreeland, R. H., Oren, A., Ventosa, A. & Kamekura, M. *Haloterrigena thermotolerans* sp. nov., a halophilic archaeon from Puerto Rico. *Int J Syst Evol Microbiol* **50** 1065-1071 (2000).
91. Ventosa, A. & Kamekura, M. *Haloterrigena*. In: *Bergey's Manual of Systematics of Archaea and Bacteria*. John Wiley & Sons, Ltd (2015).
92. Castillo, A. M. *et al. Halovivax ruber* sp. nov., an extremely halophilic archaeon isolated from Lake Xilinhot, Inner Mongolia, China. *Int J Syst Evol Microbiol* **57**, 1024-1027 (2007).
93. Hezayen, F. F., Rehm, B. H., Tindall, B. J. & Steinbuchel, A. Transfer of *Natrialba asiatica* $B1^T$ to *Natrialba taiwanensis* sp. nov. and description of *Natrialba aegyptiaca* sp. nov., a novel extremely halophilic, aerobic, non-pigmented member of the Archaea from Egypt that produces extracellular poly(glutamic acid). *Int J Syst Evol Microbiol* **51**, 1133-1142 (2001).
94. Xu, Y. *et al. Natrialba hulunbeirensis* sp. nov. and *Natrialba chahannaoensis* sp. nov., novel haloalkaliphilic archaea from soda lakes in Inner Mongolia Autonomous Region, China. *Int J Syst Evol Microbiol* **51**, 1693-1698 (2001).
95. Tindall, B., Ross, H. & Grant, W. *Natronobacterium* gen. nov. and *Natronococcus* gen. nov., two new genera of haloalkaliphilic archaebacteria. *Syst Appl Microbiol* **5**, 41-57

(1984).

96. Tapingkae, W. *et al.* *Natrinema gari* sp. nov., a halophilic archaeon isolated from fish sauce in Thailand. *Int J Syst Evol Microbiol* **58**, 2378-2383 (2008).

97. McGenity, T. J., Gemmell, R. T. & Grant, W. D. Proposal of a new halobacterial genus *Natrinema* gen. nov., with two species *Natrinema pellirubrum* nom. nov. and *Natrinema pallidum* nom. nov. *Int J Syst Bacteriol* **48 Pt 4**, 1187-1196 (1998).

98. Xin, H., Itoh, T., Zhou, P., Suzuki, K., Kamekura, M. & Nakase, T. *Natrinema versiforme* sp. nov., an extremely halophilic archaeon from Aibi salt lake, Xinjiang, China. *Int J Syst Evol Microbiol* **50 Pt 3**, 1297-1303 (2000).

99. Ruiz-Romero, E. *et al.* *Natronobacterium texcoconense* sp. nov., a haloalkaliphilic archaeon isolated from soil of a former lake. *Int J Syst Evol Microbiol* **63**, 4163-4166 (2013).

100. Kanal, H., Kobayashi, T., Aono, R. & Kudo, T. *Natronococcus amylolyticus* sp. nov., a haloalkaliphilic archaeon. *Int J Syst Bacteriol* **45**, 762-766 (1995).

101. Roh, S. W. *et al.* *Natronococcus jeotgali* sp. nov., a halophilic archaeon isolated from shrimp jeotgal, a traditional fermented seafood from Korea *Int J Syst Evol Microbiol* **57**, 2129-2131 (2007).

102. Itoh, T., Yamaguchi, T., Zhou, P. & Takashina, T. *Natronolimnobius baerhuensis* gen. nov., sp. nov. and *Natronolimnobius innermongolicus* sp. nov., novel haloalkaliphilic archaea isolated from soda lakes in Inner Mongolia, China. *Extremophiles* **9**, 111-116 (2005).

103. Xu, Y., Zhou, P. & Tian, X. Characterization of two novel haloalkaliphilic archaea *Natronorubrum bangense* gen. nov., sp. nov. and *Natronorubrum tibetense* gen. nov., sp. nov. *Int J Syst Bacteriol*, 261-266 (1999).

104. Gutiérrez, M. C., Castillo, A. M., Corral, P., Minegishi, H. & Ventosa, A.

Natronorubrum sediminis sp. nov., an archaeon isolated from a saline lake. *Int J Syst Evol Microbiol* **60**, 1802-1806 (2010).

105. Cui, H. L., Tohty, D., Liu, H. C., Liu, S. J., Oren, A. & Zhou, P. J. *Natronorubrum sulfidifaciens* sp. nov., an extremely haloalkaliphilic archaeon isolated from Aiding salt lake in Xin-Jiang, China. *Int J Syst Evol Microbiol* **57**, 738-740 (2007).

106. Ruiz-Romero, E., Valenzuela-Encinas, C., Lopez-Ramirez, M. P., de los Angeles Coutino-Coutino, M., Marsch, R. & Dendooven, L. *Natronorubrum texcoconense* sp. nov., a haloalkaliphilic archaeon isolated from soil of the former lake Texcoco (Mexico). *Arch Microbiol* **195**, 145-151 (2013).
